# Anti-Inflammatory Activity of Haskap Cultivars is Polyphenols-Dependent

**DOI:** 10.3390/biom5021079

**Published:** 2015-06-02

**Authors:** H. P. Vasantha Rupasinghe, Mannfred M. A. Boehm, Satvir Sekhon-Loodu, Indu Parmar, Bob Bors, Andrew R. Jamieson

**Affiliations:** 1 Department of Environmental Sciences, Faculty of Agriculture, Dalhousie University, Truro, NS B2N 5E3, Canada; E-Mails: mboehm@dal.ca (M.M.A.B.); Satvir.sekhon@dal.ca (S.S-L.); induparmar@Dal.Ca (I.P.); 2 Department of Plant Sciences, University of Saskatchewan, Saskatoon, SK S7N 5A8, Canada; E-Mail: Bob.bors@usask.ca; 3 Agriculture and Agri-Food Canada, Kentville, NS B4N 1J5, Canada; E-Mail: andrew.jamieson@agr.gc.ca

**Keywords:** haskap, inflammation, cytokines, polyphenols, macrophages, functional food

## Abstract

Haskap (*Lonicera caerulea* L.) berries have long been used for their health promoting properties against chronic conditions. The current study investigated the effect of Canadian haskap berry extracts on pro-inflammatory cytokines using a human monocytic cell line THP-1 derived macrophages stimulated by lipopolysaccharide. Methanol extracts of haskap from different growing locations in Canada were prepared and characterized for their total phenolic profile using colorimetric assays and liquid chromatography—Mass spectrometry (UPLC-MS/MS). Human THP-1 monocytes were seeded in 24-well plates (5 × 10^5^/well) and treated with phorbol 12-myristate 13-acetate (PMA, 0.1 μg/mL) for 48 h to induce macrophage differentiation. After 48 h, the differentiated macrophages were washed with Hank’s buffer and treated with various concentrations of test compounds for 4 h, followed by the lipopolysaccharide (LPS)-stimulation (18 h). Borealis cultivar showed the highest phenolic content, flavonoid content and anthocyanin content (*p* < 0.05). A negative correlation existed between the polyphenol concentration of the extracts and pro-inflammatory cytokines: Interleukin-6 (IL-6), tumour necrosis factor-alpha (TNF-α), prostaglandin (PGE_2_), and cyclooxygenase-2 (COX-2) enzyme. Borealis exhibited comparable anti-inflammatory effects to COX inhibitory drug, diclofenac. The results showed that haskap berry polyphenols has the potential to act as an effective inflammation inhibitor*.*

## 1. Introduction

Free radicals, produced as metabolic process mediators have a tendency to attack biomolecules like DNA, RNA, proteins and lipids [1]. The damage caused by these reactive oxygen species can vary from loss of enzyme function, increased cell permeability, and affected cell signalling causing inflammation, diabetes, and cancer [2,3,4]. Chronic inflammation is known to contribute to the risk of developing metabolic disease, which has become especially prevalent in recent years [5,6]. Inflammation is a pathophysiological response of living tissue to injuries, mediated by macrophages, leukocytes and neutrophils through oxidative species. The inflammation cascade involves various factors and enzymes, mainly cyclooxygenase (COX)-2, tumour necrosis factor-alpha (TNF-α), interferon-gamma (IFN-γ) among others. However, metabolic syndrome can be amended through addressing obesity, elevated blood sugar and cholesterol, physical exercise, and improving diet [7].

The commonly prescribed non-steroidal anti-inflammatory drugs (NSAIDs) such as aspirin (acetyl salicylic acid), diclofenac (dichloranilino phenylacetic acid) (non-specific COX inhibitor), nimesulide [N-(2-phenyloxy-4-nitrophenyl)methanesulphonamide] (COX-2 specific inhibitor) have the ability to inhibit COX enzyme which is responsible for the production of pro-inflammatory prostaglandins and prostacyclins. However, there are different undesirable effects related with these NSAID, mainly causing bleeding and ulceration in gastrointestinal tract and platelet dysfunction by blocking COX-1 derived prostanoids [8]. Hence, there is a growing interest in finding alternative food therapies, as well as new anti-inflammatory nutraceuticals derived from plant-based foods, such as fruit crops.

Phytochemicals present in functional foods such as cool climate berries offer a great hope as an alternative therapy for chronic disorders. Polyphenols in fruits have demonstrated the potential to terminate free radical reactions and many antioxidant and physiological benefits in biological systems [9]. Haskap (*Lonicera caerulea* L.), also known as blue honeysuckle, honeyberry, or sweet berry honeysuckle, is native to Siberia, China, and Japan. Haskap is fairly new to Canada, with only three major varieties in production, namely Borealis, Indigo Gem, and Tundra [10]. Along with a flavour similar to raspberries, black currants, and blueberries, haskap polyphenols also attenuates nuclear factor (NF)-kappaB dependent signaling pathway and subsequent production of proinflammatory mediators [11]. Our previous study has demonstrated the antioxidant properties and total phenolic content of the Borealis cultivar of haskap to be competitive with other berries [12], thereby suggesting its health promoting potential. Although various *in vitro* studies have evaluated blue honeysuckle extracts for their antimicrobial, anti-adherence, antioxidant effects, protective activity against ultraviolet B (UVB)-caused injury of keratinocytes (HaCaT cells), lipopolysaccharide-induced inflammation, and gingival fibroblast oxidative damage by lipopolysaccharide [11,13,14], the information on phenolic characterization and biological activity of recently introduced cultivars of Canadian haskap is limited.

Considering the previous reports, the present study was conducted to investigate the sugar, organic acid and polyphenol profiles of different haskap cultivars grown in Canada and to study their anti-inflammatory potential. In this study, human monocytes (THP-1 cells) differentiated macrophages were employed to investigate the anti-inflammatory properties of haskap berry extracts, as macrophages are predominately involved at the initial stage of inflammation process and secreting cell-signalling molecules [15].

## 2. Materials and Methods

### 2.1. Plant Material

Fruits of four haskap cultivars, Berry Blue (BL), Borealis (BR), Tundra (TN) and Indigo Gem (IG) were obtained from LaHave Forests Farm, Blockhouse, NS, Canada; two cultivars Indigo Gem (SAS-IG) and Tundra (SAS-TN) were obtained from the University of Saskatchewan, Saskatoon, SK, Canada and five genotypes, LC12, LC13, LC16, LC23, and LC47, were obtained from Agriculture and Agric-Food Canada, Kentville, NS, Canada. Berry Blue (also known as Czech No. 17) is a Czech cultivar of Russian descent with early maturity and tall plants, often used as a polliniser. The other cultivars (BR, TN, and IG) are sister seedlings bred in Saskatchewan from Blue Velvet (Kiev #3) × Blue Belle (also known as Tomichka). Kiev #3 was derived from an open pollinated plant of *L. caerulea* var. *kamtschatica* Sevast, gathered from the Kurile Islands. Czech No. 17 and Tomichka, like most cultivars of Russian descent, were derived mostly from four varieties of *L. caerulea* L*.*, but were not derived from varieties from Japan [16].

The Kentville haskap samples (*L. caerulea* L.) were selected from seedlings planted in 2007. The seed came from open pollenated fruit grown in BC, Canada in 2006 from plants originally developed by Dr. Maxine Thompson of the National Clonal Germplasm Repository in Corvallis, Oregon. The BC plants were derived from *L. caerulea* var. *emphyllocalyx* Nakai from Hokkaido. The Kentville selections flower and their fruit ripen later than the listed cultivars. The soil in Kentville was a sandy loam of the Berwick series with pH 6.8 and 2.8% organic matter. Plants grew with no pruning and minimal fertilization and 5 plants were selected based on positive fruit characteristics and designated LC-12, LC-13, LC-16, LC-23, and LC-47. Fruit from these five plants was hand-harvested for analysis on 10 July 2013. The samples were frozen at −20 °C until use.

### 2.2. Chemicals

HPLC grade methanol, acetonitrile, formic acid, Folin-Ciocalteu reagent and dimethylcinnamaldehyde were purchased from Sigma-Aldrich (Oakville, ON, Canada). The liquid chromatography standards were purchased as follows: Cyanidin-3-*O*-glucoside from Extrasynthese (Genay Cedex, France); phloridzin, phloretin, chlorogenic acid, ferulic acid and caffeic acid from Sigma-Aldrich; catechin, epicatechin, quercetin, quercetin-3*-O*-galactoside and quercitin-3*-O*-glucoside from ChromaDex, Inc. (Santa Ana, CA, USA); quercitin-3*-O*-rhamnoside, quercitin-3*-O*-galactoside and anthocyanin standards from Indofine Chemical Company (Hillsborough, NJ, USA). Hydrochloric acid, sulfuric acid, and 96-well microplates were purchased from Fisher Scientific (Ottawa, ON, Canada). Phorbol 12-myristate 13-acetate, diclofenac sodium salt, nimesulide and lipopolysaccharide were obtained from Sigma-Aldrich. COX-2 human ELISA kit was purchased from Enzo Life Sciences, Inc. (Farmingdale, NY, USA). Prostaglandin E_2_ EIA kit and nitric oxide quantification kit was purchased from Cayman Chemical Company (Ann Arbor, MI, USA). The TNF-α and IL-6 ELISA kits were purchased from BD Biosciences (San Diego, CA, USA).

### 2.3. Extraction

Frozen fruit samples (50 g) were ground and extracted with 100% methanol (250 mL) using a blender (Model HBB909, Hamilton Beach Brands Inc., Glen Allen, VA, USA) under semi-dark conditions. The extract was filtered through eight layers of cheese cloth and centrifuged at 4900× *g* for 10 min and supernatant was stored at −20 °C. Prior to performing the assays the berry extracts were evaporated of methanol under a nitrogen evaporator and stored at −80 °C until assays were performed.

#### 2.3.1. Total Phenolic Content

Total phenolic content was determined by using the modified Folin-Ciocalteu assay as described elsewhere [12]. Total phenolic content was calculated against a gallic acid calibration curve, and samples diluted when necessary to fit range. Total phenolics were expressed as gallic acid equivalents (GAE) 100 g^−1^ fresh weight (FW).

#### 2.3.2. Total Flavonoid Content

The total flavonoid assay was based on the aluminum chloride colorimetric method as described by Marinova *et al.* [17] and modified by Rupasinghe *et al.* [12]. Results were expressed as mmole quercetin equivalents (QE) 100 g^−1^ FW. The flavonoid quercetin was used as a standard to produce a calibration curve (range 50–500 mM).

#### 2.3.3. Total Anthocyanin Content

Total anthocyanin concentration (TAC) in the samples was based on the pH-differential method (AOAC method 2005.02) as previously described [18] and their concentration expressed as mg cyanidin-3-*O*-glucoside (C3G) per 100 gram FW using a molar extinction coefficient (ε) 28,000, molecular weight (MW) 484.8 for C3G.

#### 2.3.4. Total Proanthocyanidin Content

Total proanthocyanidin content was determined by the 4-dimethylaminocinnamaldehyde (DMAC) assay, as reported by Prior *et al.* [19] with modifications. DMAC reagent was prepared by adding 10 mL of acidified methanol to 0.01 g of DMAC. Then, 150 μL of DMAC reagent was added to a 96-well plate with 50 μL of berry extract and read at 640 nm. Samples were standardized against a 1000 ppm catechin stock in 5 dilutions with methanol. Total proanthocyanidin concentration was expressed as mg catechin equivalents (CE) 100 g^−1^ FW.

#### 2.3.5. LC-MS/MS Analysis of Specific Polyphenols

Total monomeric polyphenols were identified and massed by liquid chromatography—Mass spectrometry (LC-MS/MS) analysis as described elsewhere [20]. Analysis was carried out using a Waters H-class UPLC separation module (Waters, Milford, MA, USA) coupled with a Micromass Quattro micro API MS/MS system and controlled with MassLynx V4.0 data analysis system (Micromass, Cary, NC, USA). An Aquity BEH C18 (100 mm × 2.1 mm, 1.7 μm) column (Waters, Milford, MA, USA) was used.

The analysis of flavonol, flavan-3-ol, phenolic acid, and dihydrochalcone compounds was done by electrospray ionization in negative ion mode (ESI−), with a capillary voltage of 3000 V, nebulizer gas (N_2_) temperature of 375 °C, and flow rate of 0.35 mL·min^−1^. Anthocyanin compounds were analysed by electrospray ionization in positive ion mode (ESI+), with capillary voltage 3500 V, nebulizer gas at 375 °C, and flow rate of 0.35 mL·min^−1^. The cone voltage (25–50 V) was optimized for individual compounds.

#### 2.3.6. Sugars and Organic Acid Analyses

Samples were evaporated to remove methanol and dissolved in 100% deionised water prior to their filtration through a 0.45 μm nylon filter (Chromaspec, Brockville, ON, Canada) for analyses of sugars (glucose, fructose and sucrose) and organic acids (lactic, malic, quinic, citric acid) by HPLC using a Waters Alliance 2695 HPLC system connected to a Waters 2414 refractive index (RI) detector. A Rezex ROA column (250 × 4.6 mm; 8 μm; Phenomenex, Torrance, CA, USA) was used and the analysis conditions were: Temperature of 40 °C and 30 °C for column and detector, respectively; mobile phase of 0.005 N sulfuric acid in deionised water; run time of 30 min and isocratic flow rate 0.6 mL/min. An injector volume of 10 μL was used for each run. Four point calibration curves were obtained by using mixed standard solutions of sugars and organic acids at concentration range between 100 and 1000 mg/L. The results are expressed in mg 100 g^−1^ FW.

### 2.4. Cell Culture

**Figure A1:**
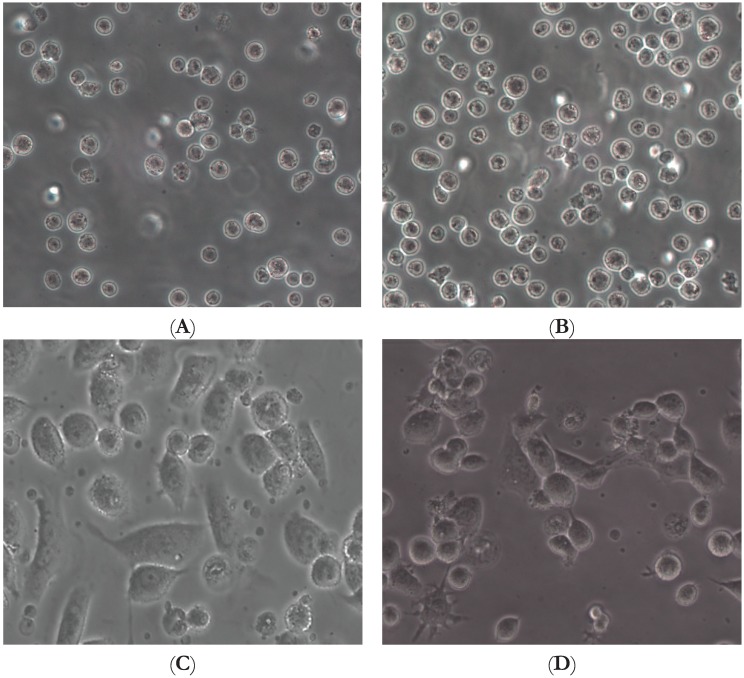
Morphology of human monocytes (THP-1) and LPS-induced macrophages. Monocytes exist in suspension phase and have spherical shape. Monocytes upon differentiation take adherent phase and form various cell shapes. The undifferentiated monocytes (**A**), (**B**) and differentiated macrophages (**C**), (**D**) are shown in the figure.

The human leukemia monocytes THP-1 (ATCC^®^ TIB202^™^) cell line was cultured at 37 °C with 5% humidified CO_2_ in Roswell Park Memorial Institute (RPMI) 1640 media with 0.05 mM of 2-mercaptoethanol and 10% fetal bovine serum. The cells were seeded in 24-well plates (5 × 10^5^/well) and treated with phorbol 12-myristate 13-acetate (PMA, 0.1 μg/ mL) to induce macrophage differentiation. Differentiated macrophages were verified by observing the cell morphology through an inverted phase contrast microscope (Figure A1) (Nikon Eclipse E 100, Nikon, Mississauga, ON, Canada). The differentiated macrophages were washed and treated with various concentrations of extracts for 4 h, followed by LPS (18 h). The supernatants were collected and stored at −20 °C for further analysis. Extracts that were used in cell culture studies were completely dried to remove solvents and reconstituted in DMSO. The controls contain the DMSO concentration equivalent to all the treatments.

#### 2.4.1. Measurement of Cell Viability

Cell viability was determined using the 3-(4,5-dimethylthiazol-2-yl)-5-(3-carboxymethoxyphenyl)-2-(4-sulfophenyl)-2H-tetrazolium (MTS) assay. THP-1 monocytes differentiated macrophages were cultured in 96-well tissue culture plate (5 × 10^4^ cells/well). Cells were treated with 50 and 100 μg/mL of haskap extract and incubated with MTS reagent for 4 h. Optical density was measured at 490 nm with a microplate reader (FLUOstar OPTIMA, BMG Labtech, Durham, NC, USA).

#### 2.4.2. Measurement of Nitric Oxide

Products of nitric oxide synthase activity were measured using Cayman’s nitrate/nitrite colorimetric assay kit (Cayman Chemical Co., Ann Arbor, MI, USA). Nitrates were converted to nitrites by nitrate reductase and total accumulated nitrites were converted to an azo compound using Griess reagent. The absorbance was read at 540 nm using the microplate reader.

#### 2.4.3. Measurement of COX-2 Activity

The COX-2 concentration was measured using human COX-2 ELISA kit provided by Enzo Life Sciences. Samples were prepared by extracting COX-2 from the cells and standards were prepared, according to instructions of the manufacturer. The plate was incubated at 37 °C for 1 h, followed by washing steps and labeled antibody was added. The plate was incubated at 4 °C for 30 min, followed by washing steps and addition of substrate solution. The reaction was stopped and absorbance was measured at 450 nm using a FLUOstar OPTIMA plate reader (BMG Labtech). The concentrations were calculated in reference to the standard curve and presented as the percentage of the inflammation control.

#### 2.4.4. Measurement of IL-6 and TNF-α

The concentrations of pro-inflammatory cytokines (TNF-α and IL-6) were measured from culture medium of control and treated cells by enzyme linked immunosorbant assay (ELISA) kit provided by BD Biosciences (Mississauga, ON, Canada). Anti-human monoclonal antibodies coated plates were developed by using detection antibodies and streptavidin-horseradish peroxidase conjugate provided by the manufacturer with each kit, according to the provided instructions. The absorbance was read at 450 nm using a FLUOstar OPTIMA plate reader (BMG Labtech). The concentrations were calculated against the standard curve and presented as the percentage of the inflammation control.

#### 2.4.5. Measurement of PGE_2_

The concentration of PGE_2_ released by LPS-stimulated macrophages was determined by acetylcholinesterase^TM^ (ACE^TM^) competitive enzyme immunoassay (EIA) kit purchased from Cayman Chemicals (Burlington, ON, Canada). The standards were prepared according to the manufacturer’s instructions. The samples (50 μL) were added to the designated wells, followed by PGE_2_-ACE tracer (50 μL) and PGE_2_-monoclonal antibody. The plates were covered for 18 h at 4 °C. The plates were developed according to the manufacturer’s instructions. The absorbance was read at 420 nm using a FLUOstar OPTIMA plate reader (BMG Labtech).

### 2.5. Statistical Analysis

All measurements were conducted in triplicate with the mean and standard deviation calculated. The normal distribution of the residuals was tested using the Anderson-Darling test. The data was analysed using ANOVA and multiple mean comparison was done using Tukey’s student range test (*t*-test) on SAS version 9.3 for Windows (SAS Institute, Cary, NC, USA). Significant differences were compared using a *p* value ≤ 0.05 for all the parameters and correlations recorded with Pearson correlation coefficients.

## 3. Results and Discussion

### 3.1. Qualitative Phenolic Composition

The total phenolic content determined by the Folin-Ciocalteu assay ranged from 634 to 1154 mg GAE 100 g^−1^ FW with the mean value of 832 mg GAE 100 g^−1^ FW (Table 1). Cultivar Borealis (BR) had the highest phenolic content and was consistent with the previous findings [12]. Tundra collected from both locations (TN and SAS-TN) had the second highest levels of phenolics (953–1015 mg GAE 100 g^−1^ FW, *p* > 0.05), while the LCs contained the lowest total phenolic content (634–849 mg GAE 100 g^−1^). Previously reported values of phenolic content of *L. caerulea* have been 428.1–622.5 mg GAE 100 g^−1^ FW by Rupasinghe *et al.* [12] and 575 to 903 mg GAE 100 g^−1^ FW by Rop *et al.* [21] that support the current data.

**Table 1:** Effect of location and cultivars on total phenolic, total flavonoid, total proanthocyanidins and total anthocyanidin content of haskap berry.

Growing Location	Cultivar	Total Phenolics (mg GAE/100 g FW)	Total Flavonoids (mg QE/100 g FW)	Total Proanthocyanidins (mg CE/100 g FW)	Total Anthocyanins (mg CGE/100 g FW)
LaHave farm	BL	755.9 ± 9.4 ^d,e,f^	1156.6 ± 121.7 ^b,c^	13.2 ± 1.0 ^c^	163.0 ± 10.1 ^c^
	BR	1154.1 ± 59.7 ^a^	1582.8 ± 140.5 ^a^	16.3 ± 0.9 ^c^	314.0 ± 2.7 ^a^
	TN	952.9 ± 18.7 ^b,c^	1260.3 ± 69.0 ^b,c^	16.0 ± 1.0 ^c^	234.4 ± 2.6 ^b^
	IG	884.3 ± 25.0 ^c,d^	1327.0 ± 12.1 ^b,c^	14.4 ± 0.5 ^c^	246.9 ± 13.7 ^b^
Kentville	LC-12	849.2 ± 28.3 ^c,d^	1035.5 ± 86.7 ^b,c^	37.2 ± 0.9 ^b^	164.9 ± 5.4 ^c^
	LC-13	796.8 ± 5.2 ^d^	997.0 ± 32.0 ^b,c^	41.0 ± 3.6 ^b^	142.5 ± 9.1 ^c^
	LC-16	664.9 ± 51.4 ^e,f^	900.7 ± 16.6 ^c,d^	52.3 ± 2.3 ^a^	70.2 ± 2.2 ^d^
	LC-23	658.1 ± 0.4 ^e,f^	956.3 ± 11.0 ^b,c^	34.2 ± 0.8 ^b^	120.2 ± 1.5 ^c,d^
	LC-47	634.4 ± 29.9 ^f^	916.5 ± 65.2 ^c,d^	47.0 ± 0.4 ^a,b^	133.4 ± 1.2 ^c^
Saskatchewan	SAS-IG	790.3 ± 68.4 ^d^	1128.5 ± 54.2 ^c,d^	19.6 ± 2.0 ^c^	246.3 ± 2.8 ^b^
	SAS-TN	1015.3 ± 78.2 ^a,b^	1428.4 ± 35.1 ^a,b^	38.5 ± 0.9 ^b^	303.2 ± 5.5 ^a^

Results represent the mean ± SD (*n* = 3), Tukey’s test, *p* < 0.05. BL, Berry Blue; BR, Borealis; TN, Tundra; IG, Indigo Gem; SAS-TN, Saskatoon Tundra; SAS-IG, Saskatoon Indigo GemGAE, gallic acid equivalents; QE, quercetin equivalents; CE, catechin equivalents; CGE, cyanidin-3-glucoside equivalents. Significant differences in each column were compared using the *p* value ≤ 0.05. ^a–f^ Different letters within rows denote significant differences between values as determined by one way ANOVA analysis.

#### 3.1.1. Total Flavonoid Content

The total flavonoid contents of the four cultivars varied from 901 to 1583 mg QE 100 g^−1^ FW with the mean value of 1154 mg QE 100 g^−1^ FW (Table 1). BR was again significantly higher in flavonoids as it was in total phenolics, which was followed by Tundra (SAS-TN and TN) and Indigo Gem (IG). Similar to the total phenolics results, the LC breeding lines had the least amount of flavonoids. Additionally, Berry Blue (BL) demonstrated a low flavonoid content of merely 1156 mg QE 100 g^−1^ FW. The present data shows about two times higher values of TFC for *L. caerulea* than one of the previous reports ranging between 594 to 699 mg QE g^−1^ FW, with Borealis containing the greatest amounts [12]. However, another finding reported much less total flavonoids (30 to 40 mg QE 100 g^−1^ FW) in blue honeysuckle [21]. This, the large variation in flavonoid content may be attributed to differences in cultivars and growing locations and conditions.

#### 3.1.2. Total Anthocyanin Content

The total anthocyanin content determined by the pH-differential method was found to have a large variation between 70 to 314 mg C3G 100 g^−1^ FW (Table 1). BR and SAS-TN were attributed with the highest levels of anthocyanins (*p* < 0.05), while the LCs was found to be significantly lower in these compounds. However, these results were lower than the previously reported values of 1470 mg C3G 100 g^−1^ for *L. caerulea* [22].

#### 3.1.3. Total Proanthocyanidin Content

The total proanthocyanidin content was found to be between 13 and 52 mg CE 100 g^−1^ FW (Table 1). Interestingly, all the LC samples showed more than twice the higher proanthocyanidin concentration than the other cultivars. While LC47 and LC16 exhibited the highest level of total proanthocyanidins, on the other hand, BL followed by BR, TN and IG showed the least content. Proanthocyanidins are considered as one of the major phenolic groups found in *L. caerulea* species [23]. However, in contrast to the previous studies [24,25] showing edible honeysuckle with very high proanthocyanidin content (195–772 mg 100 g^−1^ FW), the present results demonstrated much lower values. This discrepancy could be attributed to the difference in standards used for calibration, cultivars and geographical locations. At the time of writing, no previously published data existed stating the concentration of proanthocyanidins (for berries of genus *Lonicera*) expressed as catechin equivalents.

#### 3.1.4. LC-MS/MS Composition of Haskap Berry Extract

The phenolic characterization of haskap by LC-MS/MS is given in Table 2. In line with previous studies, cyanidin-3-*O*-glucoside followed by cyanidin-*3-O*-rutinoside were the most abundant anthocyanins observed in selected cultivars of haskap [26,27]. BR exhibited the highest cyanidin-3-*O*-glucoside concentration (76% of the total anthocyanins determined by LCMS), followed by SAS-TN (59% of the total anthocyanins). On the other hand, TN demonstrated the highest cyanidin-3-*O*-rutinoside concentration (37% of the total anthocyanins) among all the investigated cultivars. Consistent with [28], the current study also found trace amounts of petunidin and malvidin. Except for LC samples, quercetin (Q) rutinoside (also called rutin) represented the highest concentrations of 27%–88% of the total flavonols analysed by LCMS. BR presented the highest amount of 24.3 mg 100 g^−1^ FW; while LC-13 exhibited the least amount of 6.2 mg 100 g^−1^ FW. These results are in agreement with previous studies, which reported a range of 7–17 mg 100 g^−1^ FW for rutin [23,24]. This was followed by the presence of Q. arabinoside and Q. glucoside; while Q. galactoside was the least present flavonol in the selected haskap cultivars. The Kentville genotypes had lower Q. rutinoside but markedly higher Q. arabinoside (Table 2). This may prove to be a characteristic of *L. caerulea* var. *emphyllocalyx*. Among flavan-3-ol monomers, epicatechin and catechin were found most abundant in the selected haskap cultivars representing around 25%–77% and 15%–70% of the total flavan-3-ols, respectively. LC-23 and LC-16 exhibited the highest concentrations of catechin and epicatechin; while BL represented their least amount. Chlorogenic acid was the major phenolic acid observed in the Canadian haskap cultivars with amounts varying between 201–234 mg 100 g^−1^ FW. Overall, the phenolic content was found to be consistent with a previous report comparing haskap to other berries [12].

Cyanidin-3-*O*-glucoside has been reported to down-regulate the expression of inducible nitric oxide synthase (*i*NOS) in mice by suppressing the levels of proinflammatory cytokines (TNF-α, IL-6, and IL-1 β) [29], while both rutin and cyanidin-3-*O*-glucoside have shown to decrease the activity of cytokine mediated transcription factors c-Jun and nuclear factor-κB (NF-κB) [30]. Chlorogenic acid is a phenylpropanoid, produced by plants as a stress response in pathogenesis [31] and is an intermediate produced in lignin biosynthesis [32]. This compound has demonstrated an ability to reduce levels of NO, expression of COX-2 and *i*NOS, cytokines TNF-α, IL-6, IL-1 β as well as impeding the nuclear translocation of NF-κB [33]. Rutin has been reported to reduce TNF-α production and a range of interleukins in mice model [34]. It has also been reported to maintain glucose sensitivity in mice while blocking the development of insulin resistance and preventing macrophage activation [35].

**Figure 1:**
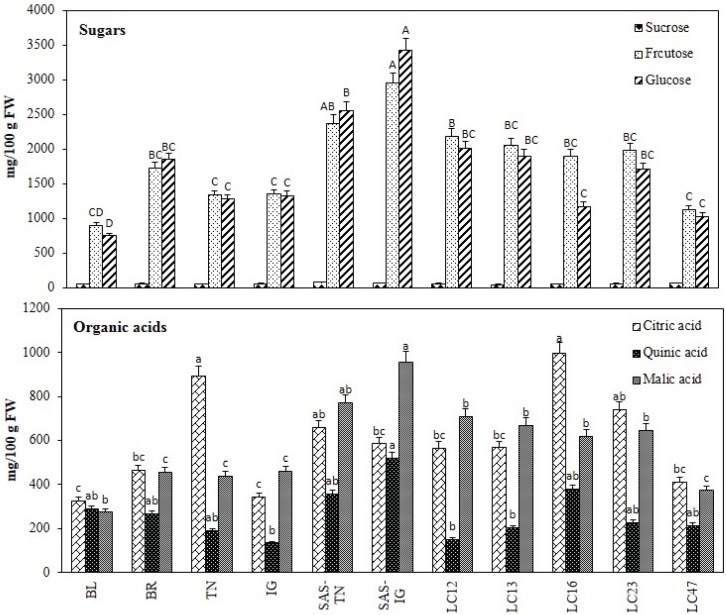
Sugar and organic acid profile of different haskap cultivars. Results represent the mean ± SD (*n* = 3), Tukey’s test, *p* < 0.05. BL, Berry Blue; BR, Borealis; TN, Tundra; IG, Indigo Gem; SAS-TN, Saskatoon Tundra; SAS-IG, Saskatoon Indigo Gem. Significance mean values at *p* value ≤ 0.05: Capital letter grouping (A–D) is for sugars and small letter grouping (a–c) is for organic acids.

**Table 2:** Concentration of polyphenols present in haskap (mg/100 g FW) as affected by cultivars and growing location.

	LaHave Farm				Kentville					Saskatchewan	
Compounds	BR	BL	TN	IG	LC-12	LC-13	LC-16	LC-23	LC-47	SAS-IG	SAS-TN
*Phenolic acids*											
Chlorogenic acid	25.6 ± 3.6	23.1 ± 2.4	26.0 ± 2.4	22.4 ± 2.5	29.7 ± 0.6	32.7 ± 3.2	23.0 ± 0.2	23.9 ± 1.3	28.6 ± 0.1	20.7 ± 1.1	33.8 ± 0.7
Caffeic acid	0.2 ± 0.0	0.1 ± 0.0	0.2 ± 0.0	0.1 ± 0.0	0.1 ± 0.0	0.1 ± 0.0	ND	0.1 ± 0.0	0.1 ± 0.0	0.1 ± 0.0	0.1 ± 0.0
Total	25.8	23.2	26.2	22.5	29.8	32.8	23.0	24.0	28.7	20.8	33.9
*Flavan-3-ols*											
EGC	0.1 ± 0.0	0.1 ± 0.0	0.1 ± 0.0	0.6 ± 0.0	0.1 ± 0.0	0.1 ± 0.0	0.1 ± 0.0	0.1 ± 0.0	0.1 ± 0.0	0.1 ± 0.0	0.1 ± 0.0
Catechin	2.5 ± 0.2	1.7 ± 0.3	3.5 ± 0.2	2.9 ± 0.3	2.1 ± 0.1	2.1 ± 0.3	3.9 ± 0.1	5.4 ± 0.0	2.1 ± 0.1	2.2 ± 0.1	3.4 ± 0.2
Epicatechin	1.2 ± 0.1	1.7 ± 0.4	0.7 ± 0.1	1.5 ± 0.1	4.5 ± 0.1	5.8 ± 0.5	7.1 ± 0.1	6.2 ± 0.1	1.5 ± 0.1	0.9 ± 0.0	0.9 ± 0.0
EGCG	0.1 ± 0.0	0.2 ± 0.0	0.2 ± 0.0	0.2 ± 0.0	0.1 ± 0.0	0.3 ± 0.0	0.1 ± 0.0	0.3 ± 0.0	0.2 ± 0.0	0.2 ± 0.0	0.2 ± 0.0
Total	3.9	3.7	4.5	5.1	6.8	8.3	11.2	12.0	4.0	3.4	4.6
*Flavonols*											
Q. galactoside	ND	0.1 ± 0.0	ND	ND	0.1 ± 0.0	0.1 ± 0.0	ND	ND	ND	ND	ND
Q. glucoside	3.6 ± 0.2	4.2 ± 0.8	1.4 ± 0.2	2.8 ± 0.2	7.3 ± 0.8	4.4 ± 0.6	3.7 ± 0.1	3.8 ± 0.1	3.7 ± 0.1	2.7 ± 0.1	4.0 ± 0.2
Q. arabinoside	2.9 ± 0.3	2.0 ± 0.3	1.4 ± 0.2	1.1 ± 0.1	11.9 ± 0.8	9.2 ± 0.8	11.4 ± 0.3	7.3 ± 0.4	10.0 ± 0.0	1.0 ± 0.0	2.9 ± 0.1
Q. rhamnoside	0.1 ± 0.0	0.2 ± 0.0	0.0 ± 0.0	0.1 ± 0.0	2.2 ± 0.2	1.2 ± 0.2	0.4 ± 0.0	0.3 ± 0.0	0.4 ± 0.0	0.1 ± 0.0	0.1 ± 0.0
Q	0.2 ± 0.0	0.3 ± 0.0	0.2 ± 0.0	0.2 ± 0.0	0.1 ± 0.0	0.1 ± 0.0	0.1 ± 0.0	0.1 ± 0.0	0.1 ± 0.0	0.2 ± 0.0	0.1 ± 0.0
Q. rutinoside	24.3 ± 1.2	16.7 ± 6.0	19.9 ± 3.0	21.6 ± 0.6	8.5 ± 0.3	6.2 ± 0.2	7.7 ± 0.6	10.9 ± 1.1	6.7 ± 0.1	19.5 ± 1.3	20.4 ± 1.3
Total	31.1	23.5	22.9	25.8	30.1	21.2	23.3	22.4	20.9	23.5	27.5
*Dihydrochalcones*											
Phloridzin	0.4 ± 0.0	0.3 ± 0.0	0.2 ± 0.0	0.3 ± 0.0	0.25 ± 0.0	0.2 ± 0.0	0.1 ± 0.0	0.2 ± 0.0	0.1 ± 0.0	0.3 ± 0.0	0.1 ± 0.0
*Anthocyanins*											
C-3-gluc	170.0 ± 10.1	140.8 ± 8.3	104.7 ± 10.3	143.9 ± 1.5	147.2 ± 5.6	107.7 ± 2.1	67.7 ± 2.7	103.4 ± 3.1	107.2 ± 0.1	138.8 ± 8.2	164.3 ± 6.2
D-3-glu	0.4 ± 0.0	0.4 ± 0.0	0.3 ± 0.0	0.4 ± 0.0	0.1 ± 0.0	0.2 ± 0.0	0.2 ± 0.0	0.2 ± 0.0	0.2 ± 0.0	0.3 ± 0.0	0.4 ± 0.0
P-3-gluc	13.7 ± 0.9	9.5 ± 0.5	6.7 ± 0.7	8.2 ± 0.3	9.9 ± 0.1	4.3 ± 0.2	3.9 ± 0.3	7.6 ± 0.3	4.9 ± 0.3	7.8 ± 0.3	14.7 ± 0.4
D-3-rutin	0.2 ± 0.0	0.1 ± 0.0	0.1 ± 0.0	0.1 ± 0.0	0.4 ± 0.0	0.2 ± 0.0	0.1 ± 0.0	0.0 ± 0.0	0.1 ± 0.0	0.0 ± 0.0	0.1 ± 0.0
C-3-rutin	39.2 ± 2.3	26.1 ± 7.5	64.9 ± 9.5	38.6 ± 2.5	19.5 ± 1.0	17.4 ± 0.6	11.8 ± 1.3	32.0 ± 0.2	22.7 ± 0.2	31.9 ± 1.3	34.6 ± 1.1
C-3-galact	0.4 ± 0.0	0.2 ± 0.0	0.8 ± 0.1	0.0 ± 0.0	0.1 ± 0.0	0.1 ± 0.0	0.0 ± 0.0	0.0 ± 0.0	0.1 ± 0.0	0.1 ± 0.0	0.4 ± 0.0
Total	223.9	177.1	177.5	191.2	177.2	129.9	83.7	143.2	135.2	178.9	214.5
**Total phenolics by LCMS**	285.1 ^a^	227.8 ^c,d^	231.29 ^c,d^	244.87 ^b,c^	244.25 ^b,c^	192.5 ^f^	209.12 ^c,d,e^	201.92 ^d,e^	188.9 ^f^	226.9 ^c,d^	280.6 ^a^

EGC, (−)-epigallocatechin; EGCG, (−)-epigallocatechin gallate; ECG, (−)-epicatechin gallate; Q, quercetin; C, cyanidin; D, delphinidin; P, peonidin; Results represent the mean ± SD (*n* = 3), Tukey’s test, *p* < 0.05. BL, Berry Blue; BR, Borealis; TN, Tundra; IG, Indigo Gem; SAS-TN, Saskatoon Tundra; SAS-IG, Saskatoon Indigo Gem; ND, Not detectable at the limit of detection of 50 ppb. Significant differences of total phenolics were compared using the *p* value ≤ 0.05. ^a–f^ Different letters within rows denote significant differences between values as determined by one way ANOVA analysis.

#### 3.1.5. Sugar and Organic Acid Profile

Citric, quinic, malic and lactic acid were quantified in the selected haskap cultivars (Figure 1). Citric acid was the predominant organic acid, accounting for 30%–58% of the total organic acid content. These results supported a recent report on *L. caerulea* that showed citric acid representing around 47% of the total organic acid content in haskap samples [23]. This was closely followed by malic acid content, which ranged between 28%–50% of the total organic acid content. The present study showed the presence of 10%–32% quinic acid with respect to the total organic acid, which was higher than reported by the previous study [23]. In contrast, lactic acid was not detected in all Canadian haskap cultivars. SAS-IG and LC-16 exhibited the highest concentration of organic acid.

The monosaccharides, glucose and fructose, predominated the haskap berries and together accounted for more than 95% of the total sugars analysed (Figure 1). Trace amounts of sucrose were also identified in the analysed fruits. Similar to the organic acid content, the highest sugar content was found in SAS-IG (6.5 g 100 g^−1^ FW; *p* < 0.05), while the lowest levels were in BL (1.7 g 100 g^−1^ FW). The content of glucose varied from 0.8–2 and 2.5–3.4 g 100 g^−1^ FW for Nova Scotia and Saskatoon berries, respectively. Similarly fructose content ranged from 0.9–2.1 and 2.3–2.9 g 100 g^−1^ FW for Nova Scotia and Saskatoon fruits, respectively. Consistent with the previously described pattern, the results showed that the cultivars with a high fructose content also had a high glucose content [23].

### 3.2. Inhibition of Inflammatory Markers by Haskap Berry Extract

**Figure 2:**
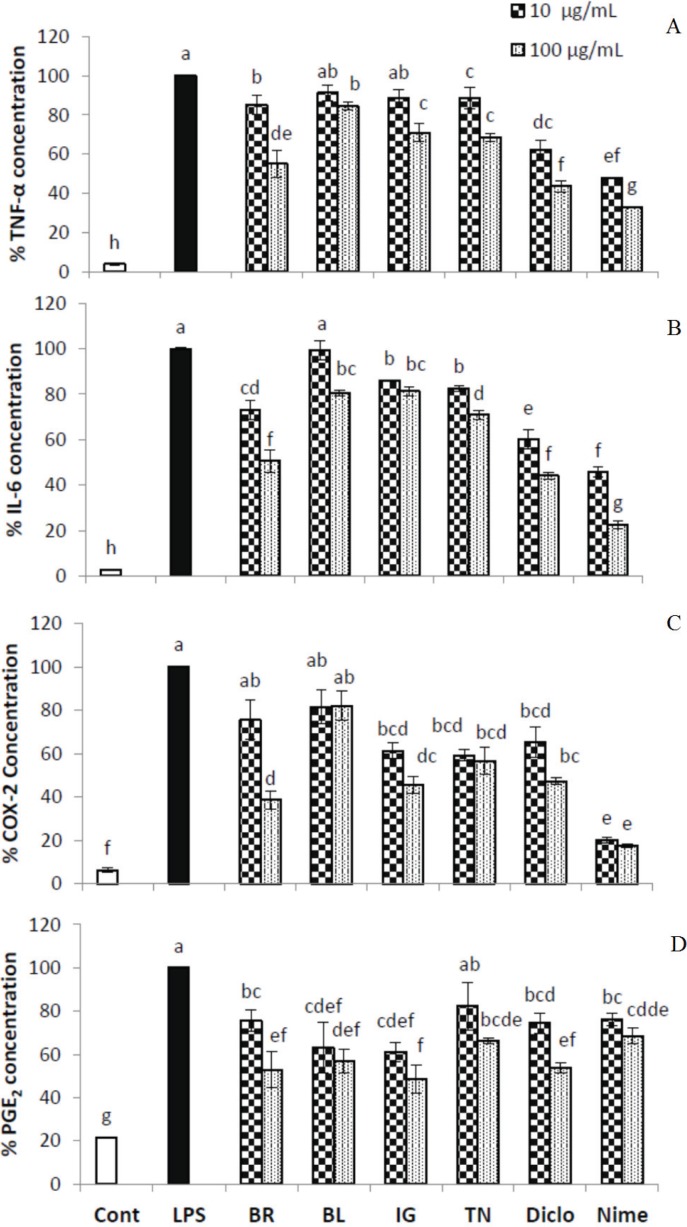
Quantification of TNF-α (**A**), IL-6 (**B**), COX-2 (**C**) and PGE_2_ (**D**) in THP-1 differentiated macrophages. The cells were pretreated with test compounds for 4 h, followed by 18 h LPS-stimulation. Results represent the mean ± SD (*n* = 3), Tukey’s test, *p* < 0.05. Cont, control; LPS, lipopolysaccharide; BR, borealis; BL, berry blue; IG, indigo gem; TN, tundra; Diclo, diclofenac; Nime, nimesulide. For each bar, different letters a-g represents significance statistical difference using Tukey’s t-test analysis (*p* ≤ 0.05). The bars with one or more same letter are not statistically different.

**Figure 3:**
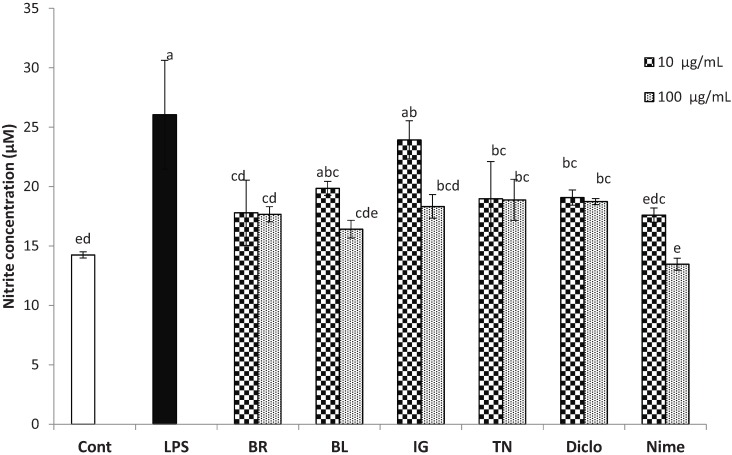
Inhibition of free-radical product (nitric oxide) by haskap berry extract in THP-1 differentiated macrophages. The cells were pretreated with test compounds for 4 h, followed by 18 h LPS-stimulation. Results represent the mean ± SD (*n* = 3), Tukey’s test, *p* < 0.05. Cont, control; LPS, lipopolysaccharide; BR, borealis; BL, berry blue; IG, indigo gem; TN, tundra; Diclo, diclofenac; Nime, nimesulide. For each bar, different letters a–e represents significance statistical difference using Tukey’s *t*-test analysis (*p* ≤ 0.05).

The working concentrations did not reduce the cell viability significantly. Following LPS stimulation, five markers of inflammation were measured and compared to two common COX inhibitors, diclofenac and nimesulide (Figure 2 and Figure 3). Cultivar BR exhibited the most consistent inhibitory effects. At a concentration of 100 μg/mL, BR extract was found to have dose dependent effects on inflammation by inhibiting cytokines TNF-α, IL-6, PGE_2_ and COX-2 to 55%, 50%, 52% and 38%, respectively (Figure 2). Furthermore, the concentrations of all five measured inflammatory markers in response to 100 μg/mL haskap extracts were comparable to the performance of diclofenac. Considering that BR was found to have the highest concentration of total phenolics (Table 1 and Table 2), the inhibition of inflammatory markers was significantly correlated with total flavonols, total anthocyanins and total phenolics (Table 3). Additionally, Borealis had the highest levels of cyanidin-3-*O*-glucoside and rutin (Table 2).

Extract of TN cultivar berries also showed significant dose dependent effects; at 10 μg/mL all markers except PGE_2_ were significantly reduced and at 100 μg/mL all parameters, TNF-α and IL-6, PGE_2_ and COX-2 were further reduced to 68%, 70% and 74%, respectively, and was as effective as diclofenac in inhibiting COX-2 (47%) and PGE_2_ (53%) (Figure 2). Cultivar IG was not an effective inhibitor for TNF-α at 10 μg/mL concentration, however, was comparable to the other three cultivars at 100 μg/mL concentration in reducing the levels of markers (Figure 2). Extract of cultivar BL was consistently the least effective at a concentration of 10 μg/mL and was not able to effectively reduce levels of COX-2 (81%), TNF-α (91%), IL-6 (99%) (*p* < 0.05) (Figure 2). Furthermore, its inhibitory effects on COX-2, TNF-α, and IL-6 at 100 μg/mL concentration were comparable to that of the other three cultivars at 10 μg/mL concentration. The dose dependent effect was not observed on NO inhibition in BR, TN extracts and diclofenac treatments (Figure 3). The extract of BL cultivar reduced NO level by 37%, followed by BR (32%), IG (30%), TN (27%), respectively, compared to LPS treatment at 100 μg/mL. Nimesulide was most effective and reduced NO level by 48% at 100 μg/mL. All extracts were comparable to diclofenac in reducing NO level (Figure 3).

**Table 3:** Correlation between phenolic measurements and five measured parameters of inflammation in LPS-induced macrophages.

Parameters	COX-2	TNF-α	IL-6	PGE_2_	NO
Phenolics	−0.781, 0.003	−0.935, 0.000	−0.896, 0.000	−0.026, 0.936	0.324, 0.304
Flavonoids	−0.742, 0.006	−0.781, 0.003	−0.723, 0.008	−0.186, 0.563	0.264, 0.406
Anthocyanins	−0.728, 0.007	−0.831, 0.001	−0.629, 0.028	0.005, 0.988	0.327, 0.300
Proanthocyanidins	−0.211, 0.511	−0.290, 0.360	−0.298, 0.348	0.459, 0.134	0.084, 0.796

Cell contents: Pearson correlation, *p*-value.

LPS is present in the cell wall of gram-negative bacteria and considered to be the most potent stimulator of monocytes and macrophages. Monocytes and macrophages play important roles at the initiation of inflammation and mobilization of the host defence against bacterial infection. LPS-stimulated macrophage activation leads to the secretion of pro-inflammatory signalling molecules known as cytokines [36]. Two common cytokines associated with inflammation are TNF-α and IL-6. TNF-α is an inflammation response protein produced in the body by macrophages, monocytes and adipocytes and endothelial cells. Long term elevated levels of TNF-α have been linked to both Type II diabetes and metabolic syndrome [37]. Reactive oxygen species can activate NF-κB by releasing inhibitor IκB-α and activated NF-κB induces the production of various cytokines [38]. The elevation of TNF-α and IL-6 levels are linked to diseases induced by inflammation and are known to stimulate endothelial cells to produce vascular cell adhesion molecules, leading to blood clots and increasing the chances of atherosclerosis [39]. Inflammatory cytokines are known to be associated with COX-2 induction, which plays a role in inflammation, as well as cell growth [40]. COX-2 is a key enzyme attributed to inducing an inflammatory response. It is known to be responsible for the production of pro-inflammatory prostaglandins (PGE_2_) at sites of injury [41]. COX-2 has been associated with a range of ailments such as heart disease, joint and muscle pain, and minor headaches. NSAIDS research to treat such symptoms accounts for a multi-billion dollar industry in the United States [42]. The inducible nitric oxide, produced by macrophages induced by TNF-α, IL-1 and IL-6, has pro-inflammatory role, whereas e-NO regulates vasodilation is anti-inflammatory in nature. Based on the growing body of evidences, the inhibition of major cytokines like TNF-α and IL-6 could be the best solution for preventing inflammation related diseases. Inhibition of inflammation appears to be most effective at the level of the two measured cytokines and COX-2 or in other words, the initial steps in the inflammation cascade. This is particularly relevant as it indicates that the haskap extract may be more effective as a preventive rather than responsive treatment to inflammation.

### 3.3. Correlation between Phenolics and Inflammatory Parameters

Overall, the correlation between levels of phenolics of the haskap and the concentrations of COX-2, IL-6, and TNF-α was found to be negative by Pearson’s correlation (Table 3). Furthermore, the flavonoids and specifically the anthocyanins showed significant negative correlation with levels of IL-6 and no significant effects were measured on levels of PGE_2_ or NO. Overall, proanthocyanidins demonstrated no statistically significant effects on the five measured markers of inflammation.

The various epidemiological studies have inversely correlated flavonoid consumption with incidence of stroke, CVD and cancer [42]. Flavonoids are renowned for their free radical scavenging abilities, which are attributed to both their physical structure and chemical activity. As an antioxidant, their primary mode of action is hydrogen atom donation by a hydroxyl group, which stabilizes the target radical to a flavonoid phenoxyl radical [43]. Quercetin, a commonly occurring flavonoid in fruits and vegetables, has been found to inhibit pro-inflammatory cytokine TNF-α by modulating the NF-κB transcription factor associated with cytokine expression [44]. Quercetin has also been reported for its benefits in diabetes [45] and hyperglycemia [46].

Anthocyanins are a sub-class of flavonoids and also function as free radical scavengers. Anthocyanins have been reported to inhibit the expression of *i*NOS responsible for producing nitric oxide [29] by suppressing the levels of pro-inflammatory cytokines earlier in the inflammatory cascade. Similarly, anthocyanins have been shown to modulate transcription factors (c-Jun and NF-κB) responsible for expressing cytokines [30]. This is an exciting prospect because the anti-inflammatory properties of haskap might not be limited to free-radical scavenging, could also be involved in gene expression of the cytokines and enzymes responsible for initiating the inflammatory cascade.

In summary, we characterized various haskap cultivars for their phenolic composition, sugar and organic profile along with anti-inflammatory properties. Among the selected haskap cultivars, Borealis was found to be the most rich in phenolic content, flavonoid content and anthocyanin content (*p* < 0.05). Cyanidin-3-*O*-glucoside, cyanidin-3-*O*-rutinoside, chlorogenic acid and rutin were polyphenols found in the highest concentrations and have been implicated in inhibiting inflammatory transcription factors in previous reports [13,14]. The phenolic profile was consistent with a previously published study of haskap [12]. We demonstrated that the extracts of haskap berry, specifically Borealis and Tundra, have markedly suppressed LPS-induced inflammation in an *in vitro* model of THP-1 derived human macrophages, when compared that with the non-specific COX inhibitor drug, diclofenac. A negative correlation was found between the polyphenols and the major inflammatory mediators. These inflammatory mediators are predominately involved at the initial stages of the inflammatory cascade and, therefore, results suggest that anti-inflammatory compounds could effectively control onset of inflammation. Overall, the results suggest that polyphenols-rich haskap berry has a potential to use as an effective functional food to control inflammation. Recently, Weidinger and colleagues [47] have demonstrated that intracellular signalling pathways mediated by NO and ROS are linked to each other via mitochondrial ROS (mtROS) and form an inducible nitric oxide synthase (iNOS)-mtROS feed-forward loop which intensifies liver failure upon acute inflammation. In general, prospective trials have shown that habitual intake of dietary polyphenols reduces the risk of dementia, stroke and neurodegenerative disorders through a wide spectrum of activities including free radical scavenging, transition metal chelation, activation of survival genes and signalling pathways, regulation of mitochondrial function and modulation of neuroinflammation [48]. Therefore, investigations will be continued to understand the possible mechanisms of haskap polyphenols in regulation of mtROS and other specific targets associated with inflammation. Further investigations are also required to find out the effectiveness of haskap berry polyphenol in animal model systems.

## 4. Conclusions

In conclusion, among the Canadian haskap cultivars assessed, Borealis exhibited the highest phenolic, flavonoid and anthocyanin contents. The polyphenol extracts of haskap could able to suppress the major pro-inflammatory cytokines including IL-6, TNF-α and PGE2 as well as COX-2 enzyme in LPS-stimulated human macrophages *in vitro*. The results suggest that haskap berry polyphenols need to be further assessed as a natural health product to control inflammation.

## References

[1] Diplock A.T., Charleux J.L., Crozier-Willi G., Kok F.J., Rice-Evans C., Roberfroid M., Stahl W., Vina-Ribes J. (1998). Functional food science and defence against reactive oxidative species. Br. J. Nutr..

[2] Antonicelli F., Parmentier M., Hirani N., Drost E., Rahman I., Donaldson K., MacNee W. (2000). LPS stimulation of IL-8 release is inhibited by thiol antioxidant at the transcriptional level in THP-1 macrophage cells. Am. J. Respir. Crit. Care Med..

[3] Mehta J.L., Rasouli N., Sinha A.K., Molavi B. (2006). Oxidative stress in diabetes, a mechanistic overview of its effects on atherogenesis and myocardial dysfunction. Int. J. Biochem. Cell. Biol..

[4] Valko M., Rhodes C.J., Moncol J., Izakovic M., Mazur M. (2006). Free radicals; metals and antioxidants in oxidative stress-induced cancer. Chem. Biol. Interact..

[5] Prescott S.L. (2013). Early-life environmental determinants of allergic diseases and the wider pandemic of inflammatory non-communicable diseases. J. Allergy Clin. Immunol..

[6] Statistics Canada Canadian Health Measures Survey: Metabolic Syndrome in Canadians. http://www.statcan.gc.ca/pub/82-625-x/2012001/article/11735-eng.htm#n1.

[7] Ford E.S., Giles W.H., Dietz W.H. (2002). Prevalence of the metabolic syndrome among us adults: Findings from the third national health and nutrition examination survey. JAMA.

[8] Gautam R., Jachak S.M. (2009). Recent developments in anti-inflammatory natural products. Med. Res. Rev..

[9] Rupasinghe H.P.V., Nair S., Robinson R., Ur Rahman A. (2014). Studies in Natural Products Chemistry.

[10] Bors B. Breeding of *Lonicera caerulea* L. for saskatchewan and Canada. Proceedings of the 1st Virtual International Scientific Conference on *Lonicera caerulea* L..

[11] Jin X.H., Ohgami K., Shiratori K., Suzuki Y., Koyama Y., Yoshida K., Ilieva I., Tanaka T., Onoe K., Ohno S. (2006). Effects of blue honeysuckle (*Lonicera caerulea* L.) extract on lipopolysaccharide induced inflammation *in vitro* and *in vivo*. Exp. Eye. Res..

[12] Rupasinghe H.P.V., Yu L.J., Bhullar K.S., Bors B. (2012). Haskap (*Lonicera caerulea*): A new berry crop with high antioxidant capacity. Can. J. Plant Sci..

[13] Palikova I., Valentova K., Oborna I., Ulrichova J. (2009). Protectivity of blue honeysuckle extract against oxidative human endothelial cells and rat hepatocyte damage. J. Agric. Food Chem..

[14] Zdarilova A., Svobodova A.R., Chytilova K., Simanek V., Ulrichova J. (2010). Polyphenolic fraction of *Lonicera caerulea* L*.* fruits reduced oxidative stress and inflammatory markers induced by lipopolysaccharide in gingival fibroblasts. Food Chem. Toxicol..

[15] Olefsky J.M., Glass C.K. (2010). Macrophages, inflammation, and insulin resistance. Annu. Rev. Physiol..

[16] Plekhanova M.N. (2000). Blue honeysuckle (*Lonicera caerulea* L.)—A new commercial berry crop for temperate climate: Genetic resources and breeding. Acta Hortic..

[17] Marinova D., Ribarova F., Atanassova M. (2005). Total phenolics and total flavonoids in Bulgarian fruits and vegetables. J. Univ. Chem. Tech. Metall..

[18] Ratnasooriya C., Rupasinghe H.P.V., Jamieson A. (2010). Juice quality and polyphenol concentration of fresh fruits and pomace of selected Nova Scotia-grown grape cultivars. Can. J. Plant Sci..

[19] Prior R.L., Fan E., Ji H., Howell A., Nio C., Payne M.J., Reed J. (2010). Multi-laboratory validation of a standard method for quantifying proanthocyanidins in cranberry powders. J. Sci. Food Agric..

[20] Rupasinghe H.P.V., Erkan N., Yasmin A. (2010). Antioxidant protection of eicosapentaenoic acid and fish oil oxidation by polyphenolic-enriched apple skin extract. J. Agric. Food Chem..

[21] Rop O., Reznicek V., Mlcek J., Jurikova T., Balik J., Sochor J., Kramarova D. (2011). Antioxidant and radical oxygen species scavenging activities of 12 cultivars of blue honeysuckle fruit. Hortic. Sci..

[22] Petrova V.P. (1986). Biochimija Dikorastuščich Plodovo—Jagodnych Rastenij.

[23] Wojdylo A., Jauregui P.N.N., Carbonell-Barrachina A., Oszmianski J., Golis T. (2013). Variability of phytochemical properties and content of bioactive compounds in *Lonicera caerulea* L. var. *kamtschatica* berries. J. Agric. Food Chem..

[24] Orincak J., Matuskovic J., Jurcak S. (2003). Possibilities of Species Lonicera caerulea in Utilization of the Secondary Metabolism in Food and Pharmaceutical Processing.

[25] Plekhanova M.N., Streltsyna S.A., Rostova N.S. (1993). Phenolic compounds in berries of *Lonicera* subsect. *Caerulea* species. Plant Res..

[26] Andersen O.M., Jordheim M., Andersen O.M., Markham K.R. (2006). The anthocyanins. Flavonoids Chemistry, Biochemistry and Applications.

[27] Gazdik Z., Krska B., Adam V., Saloun J., Jurikova T., Reznicek V., Horna A., Kizek R. (2008). Electrochemical determination of antioxidant potential of some less common fruit species. Sensors.

[28] Bakowska A.M., Marianchuk M., Kolodziejczyk P. (2007). Survey of bioactive components in Western Canadian berries. Can. J. Physiol. Pharmacol..

[29] Tsuda T., Horia F., Osawa T. (2002). Cyanidin 3*-O*-β-d-glucoside suppresses nitric oxide production during a zymosan treatment in rats. J. Nutr. Sci. Vitaminol..

[30] Karlsen A., Retterstol L., Laake P., Paur I., Kjolsrud-Bohn S., Sandvik L., Blomhoff R. (2007). Anthocyanins inhibit nuclear factor-κB activation in monocytes and reduce plasma concentrations of pro-inflammatory mediators in healthy adults. J. Nutr..

[31] Leiss K., Maltese F., Choi Y.H., Verpoorte R., Klinkhamer P.G.L. (2009). Identification of chlorogenic acid as a resistance factor for thrips in chrysanthemum. Plant Physiol..

[32] Boerjan W., Ralph J., Baucher M. (2003). Lignin biosynthesis. Annu. Rev. Plant Biol..

[33] Hwang S.J., Kim Y.W., Park Y., Lee H.J., Kim K.W. (2014). Anti-inflammatory effects of chlorogenic acid in lipopolysaccharide-stimulated RAW 264.7 cells. Inflamm. Res..

[34] Choi J.K., Kim S.H. (2012). Rutin suppresses atopic dermatitis and allergic contact dermatitis. Exp. Biol. Med..

[35] Gao M., Ma Y., Liu D. (2013). Rutin suppresses palmitic acids-triggered inflammation in macrophages and blocks high fat diet-induced obesity and fatty liver in mice. Pharm. Res..

[36] Kim Y., So H.S., Moon B.S., Youn M.J., Kim H.J., Shin Y.I., Moon S.K., Song M.S., Choi K.Y., Song J. (2008). Sasim attenuates LPS-induced TNF-alpha production through the induction of HO-1 in THP-1 differentiated macrophage-like cells. J. Ethnopharmacol..

[37] Yang J., Park Y., Zhang H., Gao X., Wilson E., Zimmer W., Abbott L., Zhang C. (2009). Role of MCP-1 in tumor necrosis factor-α-induced endothelial dysfunction in type 2 diabetic mice. Am. J. Physiol. Heart Circ. Physiol..

[38] Rahman I., Gilmour P.S., Jimenez L.A., MacNee W. (2002). Oxidative stress and TNF alpha induce histone acetylation and NF-kappaB/AP-1 activation in alveolar epithelial cells: Potential mechanism in gene transcription in lung inflammation. Mol. Cell Biochem..

[39] Thilakarathna S.H., Rupasinghe H.P.V. (2012). Anti-atherosclerotic effects of fruit bioactive compounds: A review of current scientific evidence. Can. J. Plant Sci..

[40] Vane J.R., Bakhle Y.S., Botting R.M. (1998). Cyclooxygenases 1 and 2. Annu. Rev. Pharmacol..

[41] Das U. (2001). Is obesity an inflammatory condition?. Nutrition.

[42] Knekt P., Kumpulainen J., Jarvinen R., Rissanen H., Heliovaara M., Reunanen A., Hakulinen T., Aromaa A. (2002). Flavonoid intake and risk of chronic diseases. Am. J. Clin. Nutr..

[43] Amic D., Davidovic-Amic D., Beslo D., Rastija V., Lucic B., Trinajstic N. (2007). SAR and QSAR of the antioxidant activity of flavonoids. Curr. Med. Chem..

[44] Dias A.S., Porawski M., Alonso M., Marroni N., Collado P.S., Gonzalez-Gallego J. (2005). Quercetin decreases oxidative stress, NF-kappa β activation, and iNOS overexpression in liver of streptozotocin-induced diabetic rats. J. Nutr..

[45] Nair M.P., Mahajan S., Reynolds J.L., Aalinkeel R., Nair H., Schwartz S.A., Kandaswami C. (2006). The flavonoid quercetin inhibits proinflammatory cytokine (tumor necrosis factor alpha) gene expression in normal peripheral blood mononuclear cells via modulation of the NF-kappa beta system. Clin. Vaccine Immunol..

[46] Jung M., Triebel S., Anke T., Richling E., Erkel G. (2009). Influence of apple polyphenols on inflammatory gene expression. Mol. Nutr. Food Res..

[47] Weidinger A., Mullebner A., Paier-Pourani J., Banerjee A., Miller I., Lauterbock L., Duvigneau J.C., Skulachev V.P., Redl H., Kozlov A.V. (2015). Vicious inducible nitric oxide synthase-mitochondrial reactive oxygen species cycle accelerates inflammatory response and causes liver injury in rats. Antioxid. Redox Signal..

[48] Jones Q.R.D., Warford J., Rupasinghe H.P.V., Robertson G.S. (2012). Target-based selection of flavonoids for neurodegenerative disorders. Trends Pharmacol. Sci..

